# Kgp DNA Vaccine Prevents Experimental Periodontitis

**DOI:** 10.3290/j.ohpd.b2448589

**Published:** 2021-12-18

**Authors:** Xiao Jiang, Chuanhua Li, Xin Fan, Xu Chen, Meihua Guo, Jing Lan

**Affiliations:** a Postgraduate, Department of Prosthodontics, School and Hospital of Stomatology, Shandong University and Shandong Provincial Key Laboratory of Oral Tissue Regeneration; Shandong Engineering Laboratory for Dental Materials and Oral Tissue Regeneration, Jinan, Shandong, China. *Contributed equally and share first authorship; study design, data analysis, and interpretation, drafted and critically revised the manuscript.; b Lecturer, Department of Prosthodontics, School and Hospital of Stomatology, Shandong University and Shandong Provincial Key Laboratory of Oral Tissue Regeneration; Shandong Engineering Laboratory for Dental Materials and Oral Tissue Regeneration, Jinan, Shandong, China. *Contributed equally and share first authorship; study design, data analysis, and interpretation, drafted and critically revised the manuscript.; c Lecturer, Stomatology Department, The Affiliated Hospital of Weifang Medical University, Stomatology department, the affiliated hospital of Weifang Medical University Weifang Shandong China. Study concept, data acquisition, drafted the manuscript.; d Postgraduate, Department of Prosthodontics, School and Hospital of Stomatology, Shandong University and Shandong Provincial Key Laboratory of Oral Tissue Regeneration; Shandong Engineering Laboratory for Dental Materials and Oral Tissue Regeneration, Jinan, Shandong, China. Handled the animals/attached ligature, analysed histology results, and performed statistical analysis.; e Lecturer, Department of Prosthodontics, School and Hospital of Stomatology, Shandong University and Shandong Provincial Key Laboratory of Oral Tissue Regeneration; Shandong Engineering Laboratory for Dental Materials and Oral Tissue Regeneration, Jinan, Shandong, China. Handle the animals/attached ligature, analysed histology results, performed statistical analysis.; f Professor, Department of Prosthodontics, School and Hospital of Stomatology, Shandong University and Shandong Provincial Key Laboratory of Oral Tissue Regeneration; Shandong Engineering Laboratory for Dental Materials and Oral Tissue Regeneration, Jinan, Shandong, China. Study concept and design, data interpretation, critically revised the manuscript.

**Keywords:** DNA vaccine, experimental periodontitis, IgG, Kgp

## Abstract

**Purpose:**

To investigate the prophylactic effect of lysine-specific protease (Kgp) vaccine on experimental periodontitis in mice.

**Materials and Methods:**

We constructed the eukaryotic expression plasmid pVAX1-kgp and immunised mice with the recombinant plasmid. Mice were divided into two groups and immunised with pVAX1-kgp or pVAX1 three times at 2-week intervals. Immunoglobulin (Ig)G, IgG1 and IgG2a antibodies were detected by enzyme-linked immunosorbent assay (ELISA) before and after immunisation. At the last immunisation, a silk ligature infiltrated with *Porphyromonas gingivalis* (*P. gingivalis*) was tied at the neck of the maxillary second molar to induce experimental periodontitis. Each group was euthanised after 10 days, and microcomputed tomography (micro-CT) and hematoxylin-eosin (HE) staining were used to detect the loss of alveolar bone.

**Results:**

Comparison with the pVAX1 group indicated that mice immunised with Kgp had higher levels of IgG (P < 0.05); the levels of the IgG1 were statistically significantly different (p < 0.05), and the levels of the IgG2a subtype were not significantly different. The results of micro-CT and HE staining showed that the alveolar bone loss in the pVAX1-kgp group was statistically significantly less than that in the pVAX1 group (p < 0.05). The expression of the related inflammatory factors, including interleukin-1β (IL-β), tumour necrosis factor (TNF-α) and interleukin-6 (IL-6), was lower in the pVAX1-kgp group than in the pVAX1 group.

**Conclusion:**

The Kgp DNA vaccine can enhance IgG levels in a model of experimental periodontitis, effectively activate immunity, and mitigate alveolar bone loss.

Periodontitis is a chronic infectious disease, and a main cause of tooth loss in adults. Gram-negative anaerobic bacteria and mainly *Porphyromonas gingivalis* (*P. gingivalis*)^24^ are the main pathogens of CP and are also pathogenic factors associated with cardiovascular disease (CVD), type 2 diabetes mellitus (T2DM) and Alzheimer’s disease (AD).^5,17^

The pathogenicity of *P. gingivalis* was reported to be caused by a variety of virulence factors, including gingipains, which are a family of proteases on the surface and in the secretory vesicles of *P. gingivalis.*^1^ Gingipains are one of the important independent factors of the bacteria and include arginine- and lysine-specific cysteine proteases which are known as arginine-specific gingipain (Rgp) and lysine-specific gingipain (Kgp).^12^

Gingipains account for 85% of the total extracellular proteolytic activity of *P. gingivalis*.^19^ The production of extracellular proteases by many bacterial pathogens is considered to be an important feature of the disease. These proteases are the main factors that impair the integrity and function of the host cells. In addition, the enhancement of inflammatory processes may indirectly cause pathological reactions. Kgp provides an important contribution to the pathogenicity of *Porphyromonas gingivalis*, and plays a crucial role in the survival of the bacteria and pathogenesis of periodontitis.^26^ Additionally, Kgp participates in *P. gingivalis* colonisation by binding to other bacteria in subgingival plaque and oral sulcus epithelial cells through the A1 and A3 domains of adhesin.^11^ In particular, Kgp has been shown to damage human connective tissue and plasma, including immunoglobulins; fibronectin and peptidase inhibitors, thus promoting tissue injury.^2^ Additionally, Kgp, also known as haemagglutinin,^4^ degrades a variety of haemin carrier proteins. Moreover, comparison with the wild type bacteria indicated that the growth of the Kgp mutants was significantly impaired in a specific medium, where a high molecular protein was the only source of amino acids, whereas the growth in a complex medium was unaffected. Kgp is an important enzyme for the assimilation of carbon and nitrogen in *P. gingivalis*. In animal models, Kgp inactivation resulted in a significant decrease in the virulence of *P. gingivalis*.^13^

*P. gingivalis* evades host defence mechanisms by disrupting innate immune and inflammatory responses to virulence factors. Kgp appears to be particularly relevant to the deregulation of the inflammatory response and evasion of the host defence due to the activation of the kinin cascade and conversion of the soluble complement C3 and C5 proteins.^3^ Moreover, broad spectrum antibiotic therapy rarely eradicates gingivitis, which may lead to drug resistance.^7^ The design of modern DNA vaccines generally relies on the synthesis and cloning of nucleic acids in the plasmid vectors, reducing the production costs and time.^22^ Vaccination with DNA plasmids eliminates the need to purify the proteins from infectious pathogens, improving biological safety.^10^ Plasmid DNA is also very stable at room temperature, reducing the need for cold-chain supply during transportation.^25^

Our previous experiments indicated that the pVAX1-kgp DNA vaccine enhanced immune responses and significantly delayed bone loss in the animal models of experimental peri-implantitis.^6,9^ *P. gingivalis* is the key pathogenic bacterium of periodontitis and peri-implant inflammation. In this study, we investigated the role of the Kgp DNA vaccine in periodontitis and determined the types of specific antibodies.

## Materials and Methods

### Experimental Animals

A total of 30 four-week-old male C57BL/N mice (16–18 g) were used in this experiment. The mice were randomly divided into the pVAX1-kgp vaccine group (experimental group) and the pVAX1 group (control group). The experiment was carried out after one week of adaptive housing with good lighting under clean and well-ventilated indoor conditions in a special pathogen-free environment. The room temperature was 22°C, and the light period was 12 h. Animal experiments were performed in compliance with the Animal Ethics Committee of Shandong University after the approval of the local Animal Ethics Committee, and all applicable institutional and governmental regulations concerning the ethical use of animals were followed.

### Bacterial Culture

*P. gingivalis* ATCC 33277 was cultured under anaerobic conditions (5% CO_2_, 10% H_2_ and 85% N_2_) at 37°C in brain-heart infusion (BHI) supplemented with L-cysteine (0.4 g/l), haem (5 mg/l), vitamin K1 (5 µg/ml) and yeast extract (5 g/l) (Sangon; Shanghai, China).

### Construction of the Vaccine

The DNA fragment of the Kgp gene (GenBank number: NC_010729.1) encoding the haemagglutinin domain and catalytic domain was amplified from the genome of *P. gingivalis* ATCC 33277 by PCR using the primers shown in Table 1. After digestion with NheI and XhoI, the purified gene fragment was inserted into the eukaryotic vector pVAX1 (Central Laboratory of School of Stomatology, Wuhan University, China) to obtain the pVAX1-kgp plasmid.

**Table 1:** PCR sequences of Kgp

Kgp	Forward primer	5’-GGGGGCTCGAGATGAGGAAATTATTATTGCTGATCGCG-3’ (including XhoI site GCTAGC)
	Reverse primer	5’-GGGGGTTACTTGATAGCGAGTTTCTCTACGTAAGA-3’

The structure of pVAX1-kgp was confirmed by digestion and DNA sequencing. The plasmids were isolated and purified for vaccination with an EndoFree plasmid maxi kit (Qiagen; Düsseldorf, Germany).

### Animal Immunisation Experiment

Mice were anaesthetised by isoflurane inhalation (2%, Veteasy; Shenzhen, China). Fifty micrograms of pVAX1-kgp or the same dose of pVAX1 was injected intramuscularly into the hind leg. The immunisation was performed three times at a two-week ntervals. The blood was collected retro-orbitally and used to isolate the serum (4000 rpm, 4°C, 15 min) after immunisation at 0, 2, 4 and 6 weeks. The serum was collected and stored at -80°C.

### Establishment of Animal Periodontitis Model

**Fig 1:**
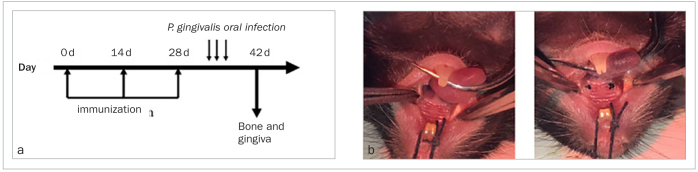
a) Experimental schedule; b) ligatures tied around the teeth.

After the last immunisation, the periodontitis model was established. Mice were anaesthetised with 10% chloral hydrate (5 mg/kg, Qilu Hospital, Jinan, China) and placed in the supine position; the limbs and head of the animals were fixed. The gingiva and mucous membrane in the mouth of the mice were observed to confirm a healthy state of the oral cavity. The mouth was sterilised with 1% iodophor, and the gingival tissue of the second maxillary molar was separated with a pointed probe. A 5-0 type 4 ligature infiltrated with *P. gingivalis* ATCC 33277 (CFU = 10^9^) was tied around the neck of the second maxillary molar, and the ligature was replaced once after 1 week (Fig 1). Two weeks later, all animals were injected with chloral hydrate for euthanasia.

### Histological Observation and Microcomputed Tomography (micro-CT)

After the animals were euthanised, the maxilla was isolated and fixed in a 4% paraformaldehyde solution. Alveolar bone loss in mice was observed by micro-CT, and the data were analysed. Some samples were subjected to HE staining of tissue sections.

### Enzyme-linked Immunosorbent Assay (ELISA)

IgG, IgG1 and IgG2a in the serum of all animals were detected by ELISA (Elabscience; Wuhan, China). The serum (2 µl) was diluted 10000 times to prepare the test samples according to instructions. The treated samples (100 µl) were added to the wells of a microplate. Fifty microlitres of enzyme-linked standard was added to the standard sample wells, and the wells were thoroughly mixed. The microplate was incubated at 37°C for 60 min. The liquid was completely removed from the wells, and the plates were washed 5 times with prediluted cleaning solution. Fifty microlitres of each of the substrates I and II was added to the wells. After the response, 50 µl of stop solution was added to each well. The OD values were read at 450 nm using a microplate reader within 30 min.

### Real-time Quantitative PCR

The mRNA expression levels of interleukin-1β (IL-β), tumour necrosis factor (TNF-α) and interleukin-6 (IL-6) in the maxilla gingiva were detected by RT-PCR two weeks after the last immunisation. Total RNA was extracted by TRIzol reagent (Vazyme; Nanjing, China), and cDNA was synthesised using a cDNA synthesis kit (Takara; Shiga, Japan). The relative expression levels were measured by the 2-ΔΔct method using β-actin as an internal control. The primers used in the experiments are listed in Table 2. All primers were constructed by Sangon Biotech (Shanghai, China).

**Table 2:** Primer sequences of IL-1β-TNF-α and IL-6

	Forward primer	Reverse primer
IL-1β	5’ GCACTACAGGCTCCGAGATGAAC 3’	5’ TTGTCGTTGCTTGGTTCTCCTTGT 3’
TNF-α	5’ ATGAGCACAGAAAGCATGA 3’	5’ ATGAGCACAGAAAGCATGA 3’
IL-6	5’ CCTCTGGTCTTCTGGAGTACC 3’	5’ ACTCCTTCTGTGACTCCAGC 3’
β-actin	5’ ACTGGGACGACATGGAG 3	5’ ACTGGGACGACATGGAG 3

### Statistical Analysis

SPSS 16.0 statistical software was used for data processing and the t-test was used for statistical analysis. p < 0.05 was considered statistically significant.

## Results

### Experimental Periodontitis Model

**Fig 2:**
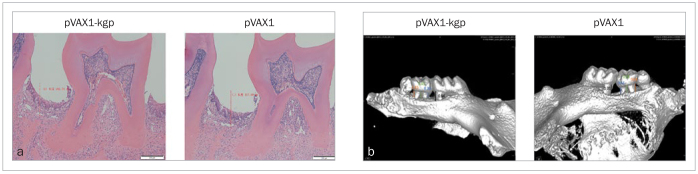
Results of HE staining and micro-CT. a) HE staining showed that bone loss was reduced in the pVAX1-kgp group (0.89-fold; p < 0.05). b) Micro-CT showed that bone loss was reduced in the pVAX1-kgp group (mesial root of the first molar: 0.63-fold*, p < 0.05; distal root of the second molar: 0.56-fold, p < 0.01; mesial root of the second molar: 0.70-fold, p < 0.05).

According to the results micro-CT and HE staining, alveolar bone loss was more pronounced in the pVAX1 group, leading to exposure of the bifurcation of the roots of the molars. The amount of alveolar bone loss in the pVAX1-kgp vaccine injection groups was less than that in the pVAX1 group (p < 0.05; Fig 2).

### Determination of Immune Response

**Fig 3:**
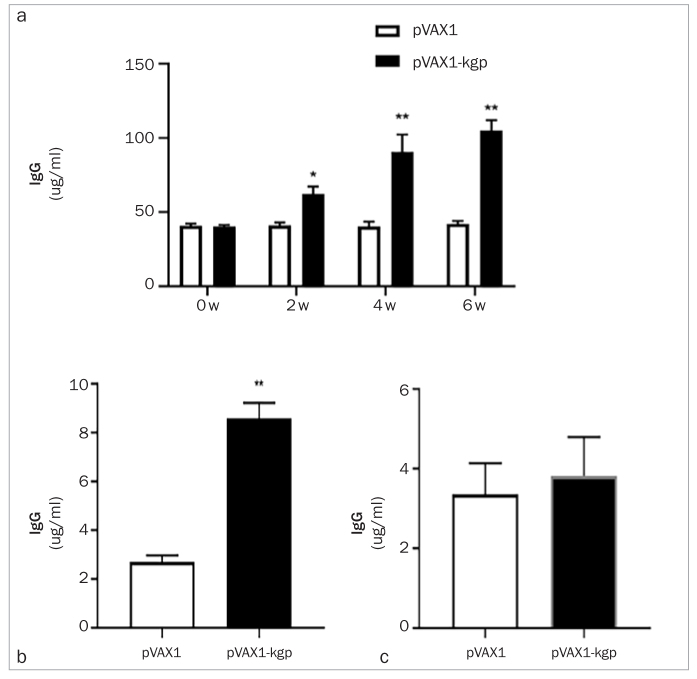
The IgG response in pVAX1 group and pVAX1 group. a) IgG levels in the pVAX1 and pVAX1-kgp groups 0, 2, 4 and 6 weeks after immunisation. b) IgG1 levels in the pVAX1 and pVAX1-kgp groups 6 weeks after immunisation. c) IgG2a levels in the pVAX1 and pVAX1-kgp groups 6 weeks after immunisation. *p < 0.05, **p < 0.01.

Before immunisation, the IgG response did not differ statistically significantly between the three subtypes (p>0.05). The IgG level in the pVAX1 group was not statistically significantly different from that detected before immunisation. After immunisation, the IgG response of the pVAX1-kgp group was statistically significantly higher than that of the pVAX1 group and higher than that detected before immunisation (p < 0.05). An increase in the immunisation time enhanced the IgG levels. The generation of IgG was dose-dependent (Fig 3a).

**Fig 4:**
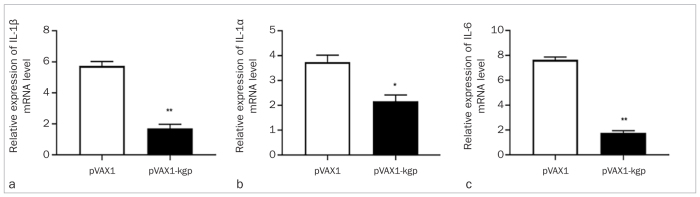
mRNA expression levels of IL-β, TNF-α and IL-6 in the maxilla gingiva. a) RT-qPCR was performed to determine the expression levels of IL-1β in the pVAX1 and pVAX1-kgp groups 6 weeks after immunisation. b) RT-qPCR was performed to determine the expression levels of TNF-α in the pVAX1 and pVAX1-kgp groups 6 weeks after immunisation. c) RT-qPCR was performed to determine the expression levels of IL-6 in the pVAX1 and pVAX1-kgp groups 6 weeks after immunisation. *p < 0.05, **p < 0.01.

The IgG subtype IgG1 level in the pVAX1-kgp group was statistically significantly higher than that in the pVAX1 group (p < 0.05). There were no differences in an increase in IgG1 between the pVAX1-kgp and pVAX1 groups (Figs 3b and 3c).

### RT-PCR Results

The mRNA expression of the relevant inflammatory factors, including IL-1β, TNF-α and IL-6, in the pVAX1-Kgp group was decreased compared with that in the pVAX1 group, clearly confirming that the pVAX1-Kgp vaccine can effectively inhibit *P. gingivalis*-induced inflammation caused by ligation with infected ligatures.

## Discussion

The pathological manifestations of rodent periodontitis include gingival inflammation, periodontal pocket formation, alveolar bone resorption and tooth loosening. Experimental and clinical reports indicated that periodontitis is associated with *P. gingivalis* infection. In this study, we constructed the pVAX1-kgp plasmid using the gingipain fragments and demonstrated that the pVAX1-kgp DNA vaccine was effective at inducing the immune response and delaying bone loss.

Periodontal disease is multifactorial,^16^ and the immune and inflammatory responses of the host to microbial invasion are the decisive factors in the development and destructive effect of the disease. Many studies have shown that helper T-cells play an important role in immune regulation.^14^ Miyachi et al^8^ immunised mice with the RgpA gene through the nasal mucosa; this immunisation effectively induced the production of salivary sIgA and blood IgG and prevented periodontal bone loss in the mice with periodontitis. The vaccine developed by our group can improve the response of Th1 cells to a certain extent. In mice, Th1 differentiation produces IgG1 antibodies, and Th2 differentiation produces IgG2a antibodies.^5^ The detection of typing antibodies in the mouse serum indicated that Th cells differentiated into Th2 cells after immunisation.

Pathirana et al^18^ demonstrated that Kgp has stronger toxic activity than does RgpA and RgpB, which also indicates that Kgp can be used as a potential target to control the occurrence of periodontal disease, by early prevention and treatment of periodontitis at the genetic level. Our previous study^9^ demonstrated that the Kgp vaccine had a stronger protective effect on alveolar bone than that of the RgpA and RgpB vaccines. In this experiment, animals from the Kgp vaccine group showed clear and stronger antibody response compared with that in the control group that eventually reduced bone loss, effectively inhibiting the development and destructive effect of periodontitis. Previous studies^9^ have shown that the RgpA and Kgp vaccines induce higher production of IgG and result in better defence against inflammatory response induced by *P. gingivalis* compared with those achieved by immunisation with the RgpB vaccine or with heat-inactivated *P. gingivalis*. Both RgpA and Kgp proteinases consist of an N-terminal propeptide region, a proteolytic domain, and C-terminal HA domains. The HA domains have highly homologous sequence.^21,23^ However, RgpB lacks the large C-terminal HA domain; thus, we hypothesised that differences due to this agglutination region may explain higher immunogenicity of RgpA and Kgp compared with that of RgpB. We plan to perform the experiments and analyses of the lectin domain in the future to verify whether this domain can be used as a targeted and short immunogen fragment.

Periodontitis is considered to have a complex source of infections. Genetic vaccine that combines antigens from various pathogens may induce an immune response with multiple antibody responses, such as the leprosy combined vaccine.^15^ The combination of independent antigen genes will be able to inhibit the development of the diseases caused by a series of pathogenic factors.^20^ Future experiments will develop a more comprehensive and effective plasmid to induce disease resistance based on these suggestions.

## Conclusion

The Kgp DNA vaccine can enhance IgG levels in a model of experimental periodontitis, effectively activate immunity, and mitigate alveolar bone loss.
